# Laparoscopic Versus Open Pyeloplasty for Primary Pelvic Ureteric Junction Obstruction: A Prospective Single Centre Study

**DOI:** 10.7759/cureus.11087

**Published:** 2020-10-22

**Authors:** Omer Farooq Rehman, Musab Umair, Amer K Hussain, Ahmad Faraz, Mohammad Iqbal, Muhammad Waqar, Muhammad Tahir, Attaur Rahman Khan

**Affiliations:** 1 Urology, Armed Forces Institute of Urology, Rawalpindi, PAK; 2 Urology, Maqsood Medical Complex and General Hospital, Peshawar, PAK; 3 Urology, California Institute of Behavioral Neurosciences & Psychology, Fairfield, USA; 4 Surgery, Cavan & Monaghan Hospital, Cavan, IRL; 5 Trauma and Orthopaedics, Leeds Teaching Hospitals NHS Trust, Leeds, GBR; 6 Orthopaedics and Trauma, Royal Shrewsbury and Telford Hospital National Health Service Trust, Shrewsbury, GBR; 7 Orthopaedics, Jinnah Postgraduate Medical Centre, Karachi, PAK

**Keywords:** ureteropelvic junction, patient reported outcomes, laparoscopic pyeloplasty, open pyeloplasty, prospective

## Abstract

Introduction

The aim of the study was to compare the clinical and patient-reported outcomes among open pyeloplasty (OP) and laparoscopic pyeloplasty (LP) patients.

Materials and methods

This was a prospective single centre, case-cohort study conducted in a tertiary care hospital with 62 patients. In both techniques, dismembered Anderson-Hynes pyeloplasty were undertaken. Post-operatively patients underwent visual analogue scale (VAS) assessment for pain, days to ambulation and comparison of the short- and long-term outcomes of the two procedures.

Results

There was no difference in the physical and functional outcomes between the two surgical approaches at 12 months period after surgery. However, patients in the laparoscopic group did report a higher rate of satisfaction at six weeks and six months’ postoperatively.

Likewise, patients in LP experienced less pain during the postoperative period (p-value <0.001), with decreased analgesic requirements. This translated into an early patient ambulation in the laparoscopic group (p-value <0.001), and a shorter hospital stay for the LP group (p-value <0.001). Moreover, follow-up ultrasound showed equal improvement of hydronephrosis among the two groups.

Conclusion

Laparoscopic and open pyeloplasty are equally effective in treating pelvic ureteric junction obstruction (PUJO), with comparable patient-reported outcomes at 12-month follow-up. However, the laparoscopic technique merits over open surgery with faster rehabilitation, a decreased postoperative pain experience and shorter hospital stay.

## Introduction

Open pyeloplasty (OP) has been the benchmark for surgical management of ureteropelvic junction obstruction (UPJO) with a success rate of 90%, as originally described by Anderson and Hynes [1]. This procedure involves making a large flank incision but implies the risk of pain, postoperative morbidity and a prolonged recovery post-surgery [2]. Marcin Polok et al. [3] conducted a 14-year-long randomized control trial, where common complications included secondary UPJO, reoperation of pyeloplasty and one patient required repeated intervention for urolithiasis.

The superlative treatment for UPJO has been a topic of debate for over a century and the effectiveness of various procedures have been investigated. Over the last 20 years, the management approaches to UPJO have expanded from OP to assorted procedures like endopyelotomy, balloon dilatation and laparoscopic pyeloplasty [4]. With the evolution of technology, laparoscopic techniques have gained popularity in urology practice and can be performed via both transperitoneal and retroperitoneal approaches [5]; however, procedures like anterograde and retrograde endopyelotomies have been reported to have inferior outcomes with a significant risk of bleeding when compared to laparoscopic procedures [6].

Schuessler et al. [7] first described laparoscopic pyeloplasty (LP) in 1993, which shortly established itself as a safe and efficacious technique under expert laparoscopic hands, with a success rate of 93% to 100%, comparable to clinical outcomes of open pyeloplasty [8]. Existing literature reports that LP has reduced the morbidity rate when compared to open pyeloplasty, with a reduction in hospital stay and less narcotic use [9].

Therefore, versatility and assurance mark LP as the superior treatment modality. Few randomized control trials have been done to compare clinical outcomes of laparoscopic and open pyeloplasty [10]. Still, they lack comprehensive data on various parameters such as postoperative pain assessment, patient satisfaction and quality of wound healing [9,10,11]. Therefore, we conducted a prospective study to assess laparoscopic vs open pyeloplasty at our tertiary care set-up to address these parameters.

## Materials and methods

Following approval from the ethical review committee, this three-year prospective study was conducted in the department of urology of a university-affiliated tertiary care hospital, from September 2016 to August 2019. The trial was registered in a publicly accessible trial registry according to the Helsinki declaration. A total of 62 patients diagnosed with primary pelvic ureteric junction obstruction (PUJO) were treated of which 30 underwent LP and 32 patients were treated by OP. After the confirmation, PUJO patients were counselled regarding surgery and were given two options, open or laparoscopic pyeloplasty. It was the patient's decision to opt for either technique at the time of preoperative counselling. Those patients who opted for open pyeloplasty were taken as a control group, whereas laparoscopic pyeloplasty patients were considered the case group.

The study included all patients diagnosed with primary PUJO and were matched for age, gender, grade of hydronephrosis and mercaptuacetyltriglycine (MAG3) scan parameters as shown in Table 1. Patients with reflux disease, single functioning kidney, duplex system, differential function <20%, previously operated PUJO or the presence of renal stones were excluded from the study.

**Table 1:** Comparison of patient demographics ^†^p-value assessed via Chi-square test; ^€^p-value assessed via independent t-test; ^£^p-value assessed via Mann-Whitney U-test. MAG3 = mercaptuacetyltriglycine.

Baseline Variables	Open Surgery (n=32)	Laparoscopic (n=30)	p-value
Mean age in years^€^	17.34±4.433	18.43±3.945	0.312
Gender^†^			
Males	18	20	0.444
Females	14	10	
Laterality^†^			
Right	12	9	0.598
Left	20	21	
Preoperative grade of Hydronephrosis^†^			
3	15	13	0.779
4	17	17	
Mean percentages of MAG3 Differential Functioning^£^	33.94±5.26	36.33±11.75	0.299
T1/2 in seconds	40.43±10.64	41.26±9.68	0.75

Preoperative workup

Patients were preoperatively evaluated by history, clinical examination, relevant blood profile (blood urea nitrogen and serum creatinine) and urine investigations (urine routine examination and culture). Diagnosis of PUJO was based upon the findings of ultrasound KUB, intravenous urography (IVU) and a MAG3 scan. Micturating cystourethrogram (MCUG) was performed in selected cases to rule our vesicoureteric reflux (VUR) disease. PUJO was defined as a symptomatic severe upper tract dilation (Grade 3/4) along with confounding evidence based on intravenous pyelography (IVP) and MAG3 scan results. Diagnostic parameters on MAG3 scan were defined as t1/2 > 20 min (time taken for the excretion of half of the radionucleotide concentration after furosemide infusion) along with confounding evidence of an obstructive type II O’Riley curve.

Surgical intervention

In both techniques, dismembered Anderson-Hyne pyeloplasty was performed. Care was taken to address a crossing aberrant lower pole vessel by repositioning the ureter ventrally.

Laparoscopic pyeloplasty

Three port laparoscopic pyeloplasty was performed transperitoneally in the modified lateral decubitus position. Pneumoperitoneum was achieved by open technique. After the mobilisation of colon, the ureter was identified and dissection performed cephalad, till the narrow PUJ segment was encountered. A percutaneous stay suture using prolene 3-0 was used to stabilize the renal pelvis, providing support for anastomosis. Excision of the segment was followed by spatulation of the ureter laterally by approximately 2 cm. Refashioning of the pelvis with excision of the redundant pelvis tissue was performed and the PUJ segment was reanastomosed with vicryl 4-0 sutures. After the posterior wall was completed, antegrade stenting was performed by passing a guidewire through a ureteric catheter using the laparoscopic suction tube. This was followed by anastomosis of the anterior wall. A 10 Fr drain was placed in all procedures through the umbilical port site after hemostatic control.

Open pyeloplasty

A flank incision with the patient in lateral position was undertaken in open pyeloplasty. After accessing the retroperitoneum, the ureter was identified and traced cranially till the PUJ segment. Traction sutures were placed on the renal pelvis followed by excision of the narrowing segment. The ureter was spatulated by approximately 2 cm and a reduction pyeloplasty was performed, where necessary. Anastomosis was undertaken using vicryl 4-0 sutures. The primary anastomotic site was sutured in interrupted fashion followed by a continuous running suture of the posterior wall. Next, antegrade stenting was performed and the anterior wall was anastomosed. After hemostatic control a 10 Fr drain was placed in the surgical bed.

 

Postoperative protocol

Patient in both groups was administered IV paracetamol 1,000 mg every six hours along with ibuprofen 800 mg IV every eight hours until oral medication could be tolerated. The visual analogue scale (VAS) was used to assess the severity of pain and a cut off > 60 was used to administer IV Tramadol 50mg, for breakthrough pain. In both procedures, the urethral catheter was ideally removed on the second post-operative day and the abdominal drain was removed prior to discharge when output was deemed insignificant. Ureteral stent was removed four weeks post-surgery.

 

Follow-up protocol

To determine clinical improvement and resolution of hydronephrosis, patients were followed up with a renal ultrasound 6 weeks post stent removal. Further imaging was arranged at six and 12 months. Success of procedure was defined as symptomatic improvement with a reduction in the degree of hydronephrosis detected on renal ultrasound (Grade <2) and improvement of MAG3 scan parameters (differential function/t1/2).

Primary outcome

Primary endpoints included quality of life assessment as per short form-12 (SF-12), a patient-reported outcome (PROM) that judge’s patient satisfaction for a given intervention. The instrument having two subscales, a physical component scale (PCS) and a mental component scale (MCS). The SF-12 form has a range of 0 to 100 with 100 indicating the highest level of patient-reported satisfaction.

Secondary outcomes

Included assessment of the postoperative pain score (VAS score), analgesic requirement, days to ambulation, success rate and comparison of the long-term outcomes of the two procedures.

Tertiary outcomes

Aimed to evaluate the mean procedural time, hospital stay and complications with respect to OP and LP. 

Statistical analysis

Statistical analysis was performed on Statistical Package for the Social Sciences (SPSS), version 23.0 (IBM, Armonk, NY), the p-value for statistical significance was kept at 0.05 with a confidence interval of 95%. Statistical tests for the comparison between the open and laparoscopic procedures were divided into continuous and categorical variables, for continuous variables a two-tailed independent t-test was and Mann-Whitney U-test were performed whereas for the categorical variables a Chi-square test and Fischer exact tests were performed.

## Results

The mean operative time was significantly shorter in the open group (90.13±12.64 vs 168.03±23.20 min) with p<0.000. Postoperative pain was analyzed using a VAS. When compared to OP, the mean postoperative analgesia requirement was significantly less in the LP group with p-value < 0.05, as shown in Table 2. Likewise, the mean length of hospital stay was comparatively shorter in LP 3.97±1.16 days, whereas stay in OP was recorded at 6.59±1.64 (p=0.000).

**Table 2:** Comparison of postoperative outcomes between open pyeloplasty and laparoscopic pyeloplasty ^††^p-value assessed via Fischer exact test; ^†^p-value assessed via Chi-square test; ^€^p-value assessed via independent t-test; ^£^p-value assessed via Mann-Whitney U-test. VAS = visual analogue scale.

	Open Surgery (n=32)	Laparoscopic (n=30)	p-value
Mean Operative time in minutes^€^	90.13±12.64	168.03±23.20	<0.001
Mean length of hospital stay in days^€^	6.59±1.64	3.97±1.159	<0.001
Average VAS for Pain on first postoperative day^£^	56.16±13.00	42.20±13.16	<0.001
Average VAS for Pain on second postoperative day^£^	45.31±12.49	31.20±12.91	<0.001
Average VAS for Pain on third postoperative day^£^	37.19±11.83	22.07±11.44	<0.001
Days to ambulation^€^	4.34±1.066	1.77±0.817	<0.001
Requirement for additional analgesia^†^	13	5	0.038
Grade of Hydronephrosis on ultrasound 6 weeks post DJ-stent removal^†^			
1	23	22	0.898
2	9	8	
Grade of Hydronephrosis on ultrasound 6 months after surgery^††^			0.774
0	25	21	
1	6	8	
2	1	1	
Grade of Hydronephrosis on ultrasound at 12 months after surgery^††^			0.306
0	30	25	
1	2	4	
4	0	1	

**Figure 1:**
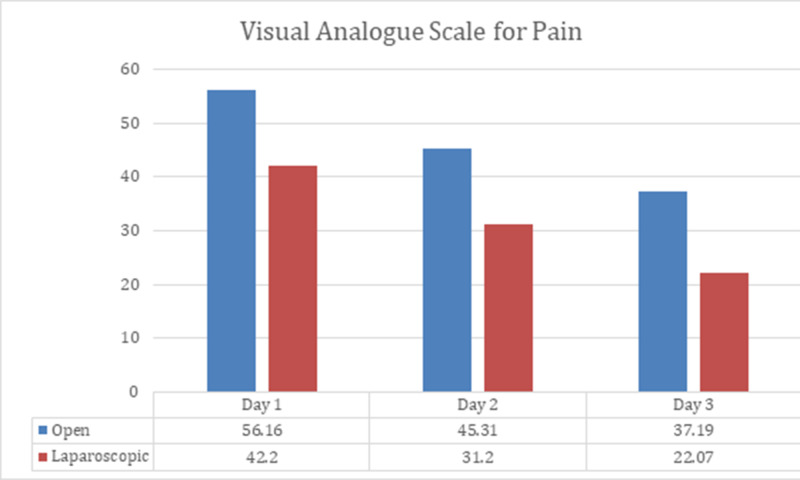
Visual analogue scale for postoperative pain

The average visual analogue for pain was recorded to be lower among laparoscopic treatment group in comparison to open surgery on the first three postoperative days, p-value < 0.01 as shown in Table 2. These findings are further elaborated in Figure 1. Patients were followed-up after six weeks, six months and 12 months, as explained below.

Six weeks

**Figure 2:**
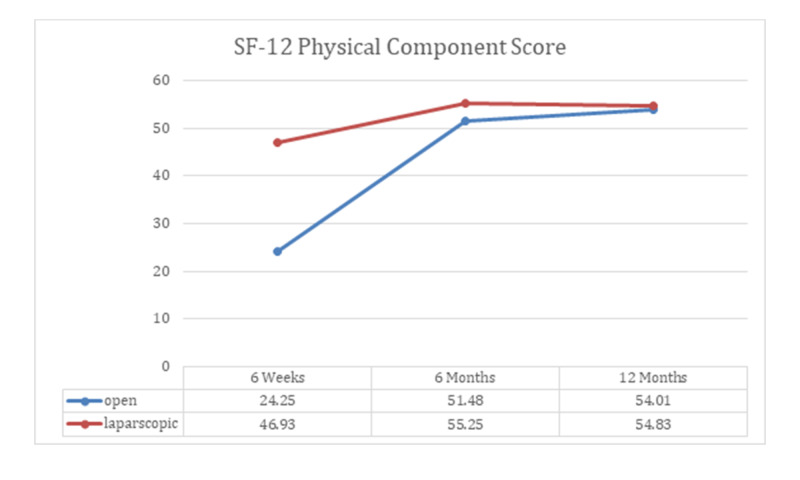
SF-12 physical component scale

**Figure 3:**
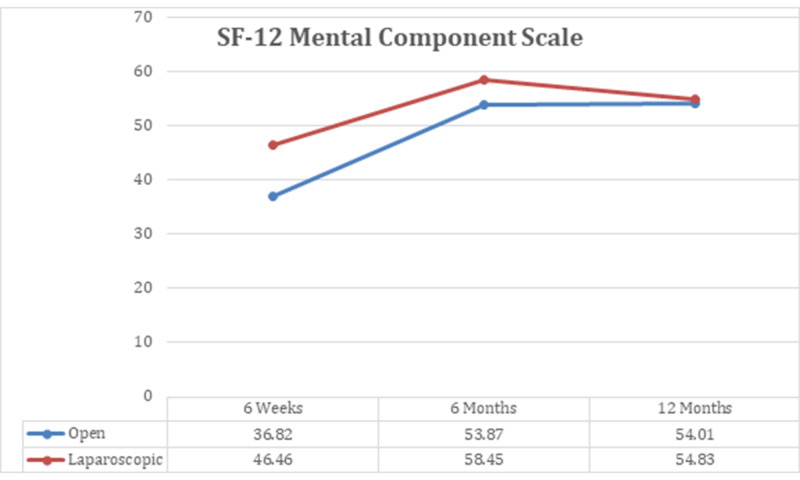
SF-12 mental component scale

After six weeks of post-DJ-stent removal, a similar number of patients were found to show improvement in hydronephrosis among open and laparoscopic pyeloplasty groups (p-value >0.05), as shown in Table 2. The SF12 for MCS and PCS was shown to be better among laparoscopic surgery group than the open pyeloplasty patients with p-value <0.000, demonstrated in Figure 2 and Figure 3.

Six months

A similar number of patients in both groups exhibited resolution of hydronephrosis at six months (Table 2), hence there was no significant difference recorded as p-value was found to be 0.774. We saw considerable difference among the two groups, when we investigated them on SF-12 MCS and PCS scales at six-month follow-up (Figures 2, 3), where p-value was noted to be 0.000.

Twelve months

When patients were screened for hydronephrosis, a similar number of cases were recorded to show improvement, as seen at six weeks and six months (Table 2). Patients undergone laparoscopic surgery were assessed to have better MCS outcome when compared to open surgery patients, p-value <0.01. However, we obtained indistinguishable PCS scores among two management groups, where p-value was found to be 0.195. Additionally, the quality of wound healing was detected to be better in laparoscopic patients, as shown in Table 3. To obtain optimum clinical outcomes, we further evaluated patients by recording their renal differential functioning through MAG3 scans; we perceived similar mean scores, i.e., OP with 41.94 ± 4.73 and LP with 40.60 ±9.10 as shown in Table 3. Finally, T1/2 was inscribed to be 9.00±4.20 among the open surgery group and 7.85±3.42 in laparoscopic patients.

**Table 3:** Primary and secondary patient-reported outcomes VAS, visual analogue scale; SF-12 MCS, SF-12 mental component scale; SF-12 PCS, SF-12 physical component scale; independent t-test was used to calculate p-value.

	Open Surgery (n=32)	Laparoscopic (n=30)	p-value
Primary Outcome			
SF-12 MCS at 12 months	53.39±3.31	56.23±2.90	0.001
SF-12 PCS at 12 months	54.01±2.295	54.83±2.66	0.195
Secondary Outcomes			
6 weeks			
SF-12 MCS	36.82±6.32	46.46±6.11	<0.001
SF-12 PCS	24.25±3.38	46.93±6.40	<0.001
6 months			
SF-12 MCS	53.87±3.44	58.45±1.95	<0.001
SF-12 PCS	51.48±3.18	55.25±2.50	<0.001
12 months			
Quality of Wound Healing VAS	72.62±4.20	86.43±4.78	<0.001
Mean percentages of MAG3 Differential Functioning	41.94±4.73	40.60±9.10	0.467
T1/2 in seconds	9.00±4.20	7.85±3.42	0.245

In the LP group, one patient was converted to OP due to intraoperative bleeding caused by iatrogenic insult to the vascular pedicle and one patient required redo-pyeloplasty due to symptomatic secondary PUJO diagnosed on MAG3 scan at nine months. Perinephric urinoma collection was observed in a single patient in each group that resolved by prolonged stenting and delayed drain removal. Two patients in the OP group and one patient in the LP group developed post-op urinary tract infection (UTI), managed conservatively with IV antibiotics. Surgical site infection was observed in a single patient of the OP group.

## Discussion

This comparative, prospective, case-control study found that there was no statistical difference in the physical and functional outcomes between open and laparoscopic pyeloplasty, at 12 months.

The pattern observed in the current study is in support of the outcomes reported by a retrospective study published by Mazhar Memon et al. [9]. Postoperative pain (VAS scores) among laparoscopic surgery has been reported to be significantly better in the present study (p-value=0.000), when compared to VAS of open pyeloplasty treatment group, these findings were authenticated by a descriptive study published by Christoph Klingler et al. [2]. Hong Mei et al. [12] promulgated a systemic review of nine studies proclaiming that OP has dramatically reduced operating time when collated with LP, where he found a p-value of 0.00001.

A similar trend was observed in this study with OP showing a reduction in operating time (p=0.000). Also, we saw significantly improved ambulation in LP group, which led to early discharge of patients and shorter hospital stay, also confirmed by Mazhar Memon et al. [9]

The purpose of pyeloplasty (OP and LP) operations in UPJO is to avoid renal complications. There is a lack of consensus on the pre-eminent type of surveillance procedure timing and frequency for the postoperative monitoring of a patient [13]. The failure of laparoscopic pyeloplasty can be early or late. In the early stage, the manifestation is often with pain, fever, or a worsening of hydronephrosis after removing the ureteral stent, whereas late findings include an excessive amount of scaring and peripelvic fibrosis [14].

Amelioration observed on MAG3 scan is a gradual process. Amling et al. [15] reviewed the transformation in pyelocalyceal dilatation after pyeloplasty among 47 renal units. He noticed that only 38% of the kidney showed improvement in the first six months after surgery, whereas 81% showed improvement by two years, as adumbrated by Park et al. [16]. We investigated patients who underwent OP after 12 months through MAG3 scan giving mean percentage was found to be 42%, whereas Shalhav et al. [17] proposed the rate of differential functioning (DRF) up to 36%.

A descriptive study published by Vishwajeet Singh et al. [18], recorded mean DRF as 33.19% among laparoscopic treatment group after one year of operation, compared to our study which reports 12-month outcome up to 40.6%. Gómez Rivas et al. [19] also reviewed differential functioning among laparoscopic pyeloplasty group and further stratified data on parameters of age (<40 and >40 years), he reported mean percentages as 47.31% (in above 40 age group) and 49.62 (in less than 40 age group).

However, we did not find any statistical significant difference in MAG3 scan improvement between the two treatment groups (p=0.467) at 12 months. MAG3 uptake and drainage were identified and defined by O'Reilly et al. [20], usually, the time consumed for clearance of 50% (t½) of the garnered radionuclide is <10 min, whereas a t½ of >20 min is indicative, but not diagnostic, of obstruction. Blum et al. [21] studied renograms of 55 patients and proposed that better (t½) has an accuracy of 83% when used for 24.5 minutes. A study published on adult laparoscopic pyeloplasty reported t½ near 14 [17], whereas the present study found t½ up to 7.85 among the laparoscopic group and 9 in the open pyeloplasty group at one year. However, this difference was not statistically significant (p-value >0.05).

Follow-up ultrasound at six months and 12 months scan showed an equivalent pattern of improvement in hydronephrosis among the two treatment groups. Song et al. [22] delineated comparable outcomes in his descriptive cohort study published in 2017, furthermore, he proposed that the use of robot-assisted laparoscopic pyeloplasty (RALP) does not make any significant difference. Additionally, conversion to open pyeloplasty is one of the significantly identified problems, Mazhar Memon et al. [9] reported the conversion of three patients from laparoscopic to open surgery. One of our patients in the laparoscopic group underwent conversion to open surgery due to intraoperative bleeding. Similarly, Punit Bansal et al. [23] also reported a case conversion, in order to achieve tension-free anastomosis.

Despite the equivalent success rate between the two groups, open pyeloplasty has the disadvantage of large flank wounds leading to surgical site infections and delayed healing [24], a similar pattern was notable in the current study, where we observed delayed wound healing in open pyeloplasty group (mean=72.62) in comparison to the laparoscopic treatment group (mean=86.43) and found the p-value < 0.001.

The 12-item Short-Form Health Survey (SF-12) is one of the most substantially used generic forms to assess patient-reported health outcome rating scales [25], which yields concise scores of physical and mental health (PCS and MCS, respectively). To the best of our knowledge, there has been no study done to report SF-12 scores with MCS and PCS. Our assessment reveals that there was a significant difference (p-value < 0.001) observed at six weeks and six-month follow-up, among the two treatment groups, when they were investigated on the mental and physical component scales of SF12. Additionally, at 12-month follow-up, we also recorded significant outcomes on MCS with p-value of 0.001. However, no difference was noted on physical component scales after 12 months, and this could be due to the possibility of overall improvement in the physical condition of the patient after surgery.

Limitations and strengths

Our study is limited due to its sample size, non-randomized design and lacking long-term outcomes after 12 months of the procedure. Secondly, cost-effectiveness between the two procedures was not compared. 

On the other hand, this study provides an account of the patient satisfaction between the surgical groups that are not available in the published literature for PUJO. Furthermore, this study compares the outcomes of PUJO among adolescent and adult patients, whereas PUJO in developed countries is treated in the paediatric age group, primarily due to good antenatal screening. The findings of this study can provide valuable insight into the management of PUJO in adolescents and adult population.

## Conclusions

In conclusion, laparoscopic and open pyeloplasty are equally effective in treating PUJO, with comparable patient-reported outcomes at 12-months follow-up. However, the laparoscopic technique merits over open surgery with faster rehabilitation, a decreased postoperative pain experience and a shorter hospital stay.

## References

[1] O'Reilly PH, Brooman PJ, Mak S (2001). The long-term results of Anderson-Hynes pyeloplasty. BJU Int.

[2] Christoph Klingler C, Remzi M, Janetschek G, Kratzik C, Marberger MJ (2003). Comparison of open versus laparoscopic pyeloplasty techniques in treatment of uretero-pelvic junction obstruction. Eur Urol.

[3] Polok M, Apoznański W (2017). Anderson-Hynes pyeloplasty in children - long-term outcomes, how long follow up is necessary?. Central Eur J Urol.

[4] Dinlec CZ, Smith AD (2000). Editorial comments: current techniques for treating ureteropelvic junction obstruction. Braz J Urol.

[5] Srinivas KK, Uppin IV, Nerle RB (2011). A Prospective Randomized Controlled Trial Complains Open Pyeloplasty and Laparoscopic Pyeloplasty for Ureteropelvic Junction Obstruction (UPJO): Subjective Outcome. J Clin Diagn Res.

[6] Rassweiler JJ, Subotic S, Feist-Schwenk M (2007). Minimally invasive treatment of ureteropelvic junction obstruction: Long-term experience with an algorithm for laser endopyelotomy and laparoscopic retroperitoneal pyeloplasty. J Urol.

[7] Schuessler WW, Grune MT, Tecuanhuey LV, Preminger GM (1993). Laparoscopic dismembered pyeloplasty. J Urol.

[8] Stein RJ, Inderbir SG, Desai MM (2007). Comparison of surgical approaches to ureteropelvic junction obstruction: endopyeloplasty versus endopyelotomy versus laparoscopic pyeloplasty. Curr Urol Rep.

[9] Memon M, Biyabani SR, Ghirano RA, Aziz W, Siddiqui KM (2016). Is laparoscopic pyeloplasty a comparable option to treat ureteropelvic junction obstruction (UPJO)? A comparative study. J Pak Med Assoc.

[10] Autorino R, Eden C, Gettman M (2014). Robot-assisted and laparoscopic repair of ureteropelvic junction obstruction: a systematic review and meta-analysis. Eur Urol.

[11] Bansal P, Gupta A, Mongha R (2008). Laparoscopic versus open pyeloplasty: comparison of two surgical approaches- a single centre experience of three years. J Minim Access Surg.

[12] Mei H, Pu J, Yang C, Zhang H, Zheng L, Tong Q (2011). Laparoscopic versus open pyeloplasty for ureteropelvic junction obstruction in children: a systematic review and meta-analysis. J Endourol.

[13] Faure A, London K, Smith H (2016). Early mercaptoacetyltriglycine(MAG‐3) diuretic renography results after pyeloplasty. BJU Int.

[14] Chiancone F, Fedelini M, Pucci L, Meccariello C, Fedelini P (2017). Laparoscopic management of recurrent ureteropelvic junction obstruction following pyeloplasty: a single surgical team experience with 38 cases. Int Braz J Urol.

[15] Amling CL, O’Hara SM, Wiener JS, Schaeffer CS, King LR (1996). Renal ultrasound changes after pyeloplasty in children with Ureteropelvic Junction obstruction: long-term outcome in 47 renal units. J Urol.

[16] Park K, Baek M, Cho SY, Choi H (2013). Time course of hydronephrotic Changes following unilateral pyeloplasty. J Pediatr Urol.

[17] Shalhav AL, Mikhail AA, Orvieto MA (2007). Adult stentless laparoscopic pyeloplasty. JSLS.

[18] Singh V, Garg M, Sharma P, Sinha RJ, Kumar M (2015). Mini incision open pyeloplasty - improvement in patient outcome. Int Braz J Urol.

[19] Gómez Rivas J, Gregorio SA, Eastmond MP (2014). Renal function recovery after laparosocopic pyeloplasty. Central Eur J Urol.

[20] O'Reilly P, Aurell M, Britton K, Kletter K, Rosenthal L, Testa T (1996). Consensus on diuresis renography for investigating the dilated upper urinary tract. Radionuclides in Nephrourology Group. Consensus Committee on Diuresis Renography. J Nucl Med.

[21] Blum ES, Porras AR, Biggs E (2018). Early detection of ureteropelvic junction obstruction using signal analysis and machine learning: a dynamic solution to a dynamic problem. J Urol.

[22] Song SH, Lee C, Jung J (2017). A comparative study of pediatric open pyeloplasty, laparoscopy-assisted extracorporeal pyeloplasty, and robot-assisted laparoscopic pyeloplasty. PLoS One.

[23] Bansal P, Gupta A, Mongha R (2011). Laparoscopic versus openpyeloplasty: comparison of two surgical approaches- a single centre experience of three years. Indian J Surg.

[24] Calvert RC, Morsy MM, Zelhof B, Rhodes M, Burgess NA (2008). Comparison of laparoscopic and open pyeloplasty in 100 patients with pelvi-ureteric junction obstruction. Surg Endosc.

[25] Shenkman E, Muller K, Vogel B (2015). The wellness incentives and navigation project: design and methods. BMC Health Serv Res.

